# AGING, DISASTERS, AND THE ENVIRONMENT: ROLE OF CLIMATE CHANGE IN DISASTER PREPAREDNESS AND RESPONSE

**DOI:** 10.1093/geroni/igz038.1989

**Published:** 2019-11-08

**Authors:** Judith Robertson R Phillips, Melissa L Cannon, Zhen Cong

**Affiliations:** 1 California State University San Marcos, San Marcos, California, United States; 2 Western Oregon University, Monmouth, Oregon, United States; 3 School of Social Work, University of Texas at Arlington, Arlington, Texas, United States

## Abstract

Continuing changes in the world’s climate put the older adult population more at risk than other age groups. Multiple circumstances such as disasters and environmental events brought about by climate change impair the ability of older adults to maintain healthy physical and psychological well-being. Unfortunately, during these times, public and individual emergency preparation plans often fall short of having the needed resources to reach out and offer assistance to older adults. Co-sponsored by the Environmental Gerontology and Disasters and Older Adults Interest Groups, four presenters will highlight multiple circumstances where older adults face serious physical and psychological consequences as a result of climate change issues: 1) the loss of electrical power and its impact on hypothermia and hyperthermia in older adults, 2) the loss of basic public health services after Hurricane Maria and its contribution to older adults’ reduced psychological well-being including increased levels of suicide, 3) homeless older adults experiencing a disaster and their resulting increased need for resources, and 4) older adults in the path of violent tornadoes and their ability to cope on their own. These presenters will discuss actions, interventions, and development of emergency disaster preparation plans that would be beneficial for older adult response and recovery. The fifth paper will review the theoretical backgrounds of and previous studies on Anthropocene with a focus on climate change policy and practice. Collectively, these papers will address various means by which older adults are affected by climate change and optimal practices to speed recovery.

